# Supervised deep learning with vision transformer predicts delirium using limited lead EEG

**DOI:** 10.1038/s41598-023-35004-y

**Published:** 2023-05-16

**Authors:** Malissa A. Mulkey, Huyunting Huang, Thomas Albanese, Sunghan Kim, Baijian Yang

**Affiliations:** 1 College of Nursing, University of South Carolina, Columbia, SC USA; 2 Department of Computer and Information Technology, Purdue University, Lafayette, IN USA; 3 Department of Engineering, University of East Carolina, Greenville, NC USA

**Keywords:** Biotechnology, Computational biology and bioinformatics, Neuroscience, Physiology, Biomarkers, Medical research, Neurology

## Abstract

As many as 80% of critically ill patients develop delirium increasing the need for institutionalization and higher morbidity and mortality. Clinicians detect less than 40% of delirium when using a validated screening tool. EEG is the criterion standard but is resource intensive thus not feasible for widespread delirium monitoring. This study evaluated the use of limited-lead rapid-response EEG and supervised deep learning methods with vision transformer to predict delirium. This proof-of-concept study used a prospective design to evaluate use of supervised deep learning with vision transformer and a rapid-response EEG device for predicting delirium in mechanically ventilated critically ill older adults. Fifteen different models were analyzed. Using all available data, the vision transformer models provided 99.9%+ training and 97% testing accuracy across models. Vision transformer with rapid-response EEG is capable of predicting delirium. Such monitoring is feasible in critically ill older adults. Therefore, this method has strong potential for improving the accuracy of delirium detection, providing greater opportunity for individualized interventions. Such an approach may shorten hospital length of stay, increase discharge to home, decrease mortality, and reduce the financial burden associated with delirium.

## Introduction

Delirium is an acute syndrome manifested by a change in global cognitive function that includes either disorganized thinking or an altered level of consciousness^1^. Delirium occurs in as many as 80% of critically ill older adults and is associated with worse long term cognitive outcomes^2,3^. For more than 20 years, at least 10 national and international health care professional organizations have included routine delirium screening in clinical practice guidelines^4–6^. Despite these recommendations and the availability of more than 40 validated screening tools, less than 10% of clinicians report routinely screening for delirium^4,7^. In the ICU environment, many patients are unable to participate in delirium screening, such as those in a comatose or deeply sedated state, and are therefore, untestable. Even when these tools are used, delirium remains difficult to recognize and therefore frequently underdiagnosed and undertreated. As the duration and severity of delirium increases it becomes increasingly more difficult to treat. As a result, delirium is associated with a one-year increase in economic burden of more than $44 K/patient, making it a global public health crisis^8^.

**Figure 1:**
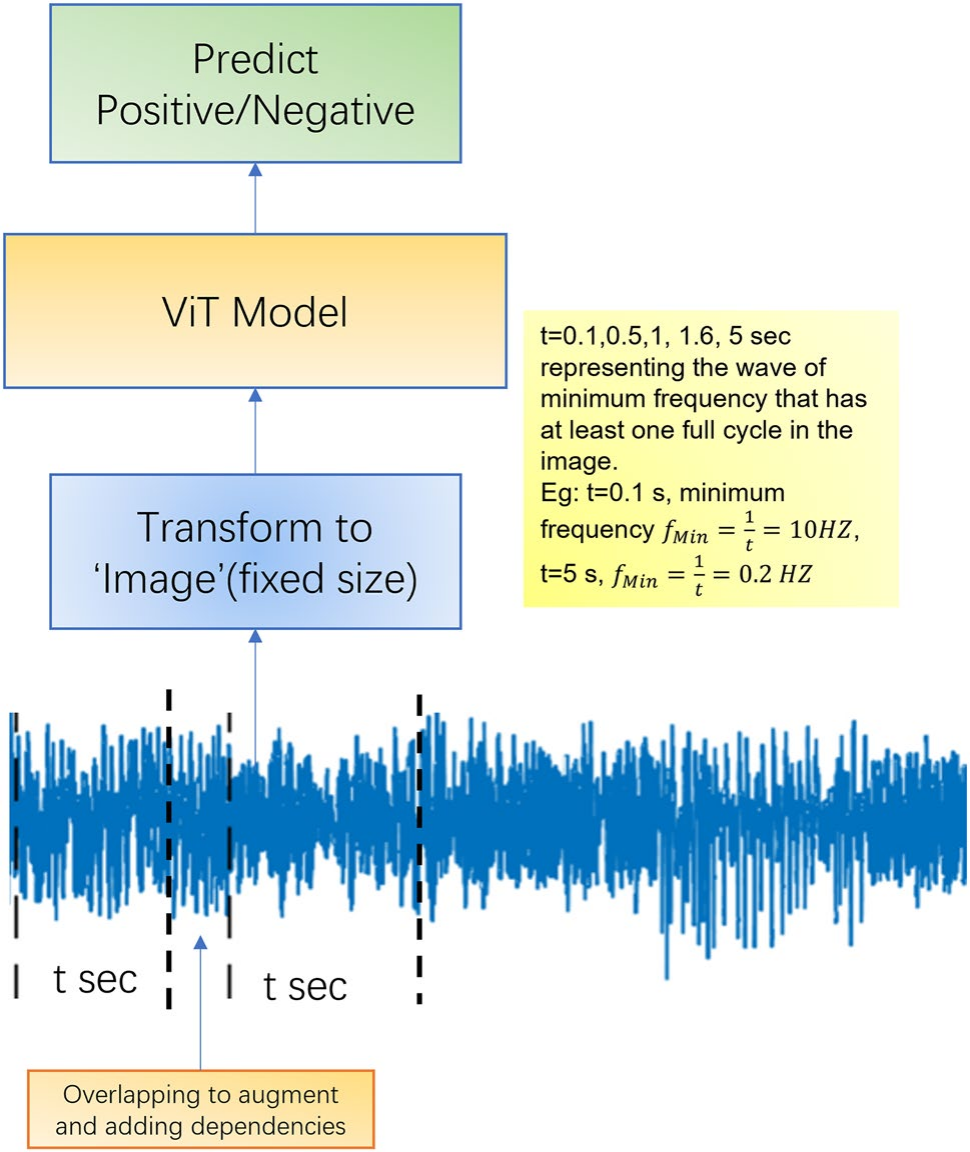
Our working pipeline.

The electroencephalogram (EEG) is a representative signal with information describing the condition of the brain. The shape, amplitude, and oscillation speed of EEG waveforms help describe the condition and assist with diagnostics as shown in Fig. 1. Use of EEG for delirium detection was first identified in the 1940’s. Romano and Engel identified slowing of EEG with increases in sleep and decreases in awake waves when delirium was present^9,10^. Thus, delirium has been reliably identified by examining changes in neural activity using the EEG. Unfortunately, the significant cost associated with technological set up and the need for expert analysis has prevented the use of EEG for delirium detection in the clinical environment^11,12^.

**Step 1** Extract subsets from the data, each subset has record t sec. Split these subsets into training/testing set.

**Step 2** Transform subsets into ‘images’ (*).

**Step 3** Use these ‘images’ to feed ViT model.

More recently, user friendly handheld EEG devices with recording accuracy equivalent to traditional EEG that are programmed with rapid response analytic methods such as machine learning have become available^13^. These devices offer rapid setup by anyone with limited training, thus providing rapid EEG data (thus rapid response EEG) within minutes, unlike traditional EEG that can take up to an hour to setup and requires specially trained staff. To evaluate EEG waveforms, signal parameters are extracted and analyzed using computer based statistical algorithms. For example, nonlinear time series analysis offers insight into the dynamic nature and variability of brain signals^14^. With the development of an algorithm capable of accurate predictive detection, newer EEG devices may provide a feasible physiologic method to support clinicians with delirium detection.

The most widely used machine learning technique in current research and practice is called supervised machine learning where the ground truth is known to the researchers and is labeled in the training dataset. Sophisticated deep neural networks are often adopted and optimized to fit the needs of specific learning tasks in real world scenarios. While deep-learning-based approach often outperforms the traditional statistical machine learning algorithms in prediction accuracy, its Blackbox nature often makes the model impractical to use in the medical field. Additional modules are often needed to help explain why or how the decision was made by the deep learning algorithms.

The supervised deep learning model used in this study was Vision Transformer (ViT)^15–17^. The ViT model leveraged the cutting-edge Transformer architecture that has revolutionized the field of Natural Language Processing (NLP). It should be noted that EEG data are typically sequential in nature. Therefore, sequential models, such as BERT^18^ and Fractional Dynamics Foster Deep Learning were often applied in the literature. However, EEG data in tabular format comprises a combination of waves with varying frequencies, which makes the conventional sequential approaches less effective^19^. The EEG data in wave image format contains both temporal data and visual patterns. Due to its outstanding performance in computer vision related problems, ViT has been applied in recent image classification tasks and improvements in performance have been seen over traditional CNN based frameworks^20^. In this work, we investigate how ViT can be used to gain intelligence from EEG data. We show that applying ViT in time domain wave image format is much better than applying ViT in frequency domain.

To make the Transform model work for image classification tasks, the core idea is to slice an image into a matrix of $n \times n$ sub-images. These sub-images are then treated as sequential data so that the self-attention mechanism can be applied to measure the relationship between pairs of sub-images. The benefit of ViT is the ability to maintain spatial information as well as temporal information. EEG data are sequential and have a spatial relationship, making the ViT an ideal model for this analysis. This paper describes how machine learning using Vision Transformer can serve as an electronic means for detection of delirium, at minimal risk and low cost.

## Methods

This is the first prospective proof-of-concept pilot study using a rapid response EEG device providing data from all cerebral lobes, and a supervised deep learning method (Vision Transformer) to evaluate EEG for the presence of delirium in critically ill patients.

The study (UMCIRB 17-001900 MIND) was reviewed and approved by the East Carolina and Vidant Medical Center Institutional Review Board (UMCIRB) on March 13, 2018. Written informed consent was obtained from the participant’s legally authorized representative prior to any research activities. All research procedures were conducted in accordance with the ethical standards set by the UMCIRB IRB and the Helsinki Declaration of 1975.

### Setting/sample

The protocol for this study was previously published in RINAH^21^. In brief, patients meeting inclusion and exclusion criteria were recruited from three intensive care units (cardiac, medical, and surgical ICU) in a large rural academic medical center in North Carolina between March 2019 and March 2020. All participants were at least 50 years old and required mechanical ventilation for greater than 12 h who were English speaking for whom written informed consent from the legally authorized representative was obtained. Exclusion criteria included acute brain injury, seizures, or condition preventing participation in the delirium screening. Consent was obtained prior to enrollment from the legally authorized representative because participants were unable to self-consent.

Each day, the patient was assessed for the ability to participate in a delirium screening determined using the Richmond Agitation Sedation Scale (RASS)^22,23^. The RASS is a 10 level scale (+ 4 “combative” to − 5 “unarousable”) with excellent inter-rater reliability (r = 0.956, lower 90% confidence limit = 0.948; κ = 0.73, 95% confidence interval = 0.71, 0.75)^22,23^. A RASS score of − 2 or higher (able to open eyes for > 10 s to voice) met eligibility.

Demographic and clinical characteristics were obtained from the electronic medical record (EMR).

### Measures

#### Bedside behavioral assessment for delirium

The Confusion Assessment Method for the Intensive Care Unit (CAM-ICU) is a modified version of the CAM that was developed to assess mechanically ventilated and non-verbal patients in the ICU^24,25^. The CAM-ICU is based on the gold standard for delirium identification, the Diagnostic and Statistical Manual for Mental Disorder IV (DSM-IV) and one of two delirium screening tools recommended for use by the Society of Critical Care Medicine (SCCM)^1,4,26^. The CAM-ICU requires patient participation to identify four key features of delirium including an (a) acute onset or fluctuation in mental status within the previous 24 h., (b) inattention, (c) altered level of consciousness [Richmond Agitation Sedation Scale (RASS) ≠ 0], and (d) disorganized thinking^24,25^. When used in research the CAM-ICU has high sensitivity and specificity, 93 and 98% respectively and high inter-rater reliability at k.0.79.

#### Physiologic assessment for delirium

Rapid response EEG headbands that circumscribe the head were applied to each participant daily. Accuracy of placement is based on location of the headband fastener in the center of the forehead, electrodes are numbered 1–10, headband connects to the recorder at the hairline on the back of the head, EEG waveforms are immediately visualized on the EEG recorder and the recorder identifies quality of connection (e.g., impedance) using a color-coded diagram (green = low impedance/red = high impedance) of the headband. EEG monitoring occurred for 2 h each evening between 5 pm and 9 pm (1700–2100) for four days or ICU discharge. After one hour of EEG monitoring, the participant was assessed for delirium by the research team using the Confusion Assessment Method for the ICU (CAM-ICU). To be considered delirium positive using the CAM-ICU the participant must have at least three of the core components of delirium including an acute change in baseline mental status, exhibit inattention, and have either an altered level of consciousness or disorganized thinking.

#### Processing the EEG data

Prior to analysis, EEG data were processed to remove artifacts such as muscle movement in the face and interference from nearby devices such as ventilators and cardiac monitors. To do this, high and low frequencies are removed using filters. Data are then re-referenced to estimate physiological noise and divided into multiple discrete time periods called epochs. After pre-processing, EEG data is further “cleaned” using Individual component analysis (IDA) to remove noises and generate features needed for machine learning algorithms. Component analysis is a widely accepted method to clean data by separating artifact from data derived from cortical processes^14,27^. The benefit of independent component analysis with higher order statistics is the ability to simply subtract artifacts by directly examining the independent components of the data.

#### Machine learning

EEG analytic techniques across studies have varied. Therefore, both traditional and three machine learning methods were used initially to analyze these data, specifically random forest (series of decision trees), stepwise linear discriminant analysis (removes variables that do not help classify the data, in this case delirium−/delirium+), and support vector machine (computer builds a model to provide the greatest difference between categories, in this case delirium−/delirium+). Due to challenges with feature selection, a supervised deep learning method, i.e., Vision Transformer, was primarily used in this analysis. It is observed in many deep learning applications that sophisticated data processing techniques and feature engineering are often not needed because the deep neural networks can learn those subtle features directly from the input data. Two types of data were therefore studied. The first type of input data was preprocessed (muscle movement and device interference removal) and IDA cleaned while and the second type of input received only preprocessing with no IDA cleaning. Data were extracted every 4 ms from the EEG devices and each data sample contains the reading of eight sensors.

**Figure 2:**
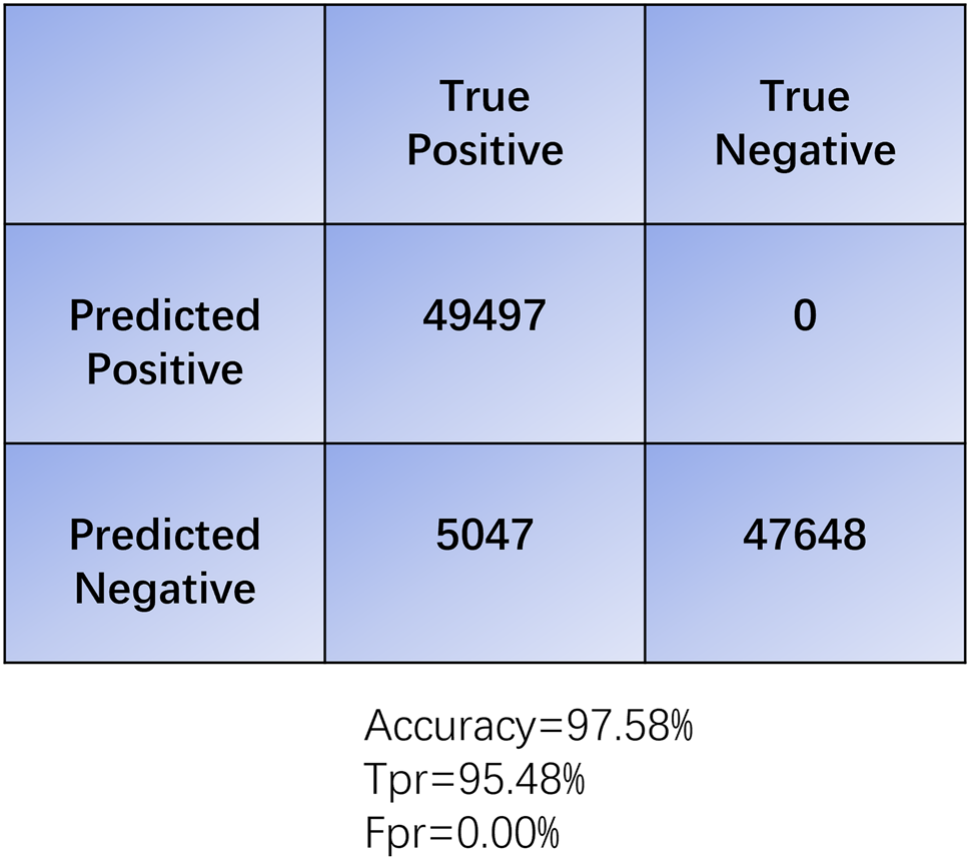
The confusion matrix for the experiment.

A continues of number of rows of data is organized into a data slice, which is an $8 \times n$ array, where $n$ represents the number of rows. These arrays are resized to $224 \times 224$ using Bilinear interpolate and are treated as images to feed into the ViT model, as shown in Fig. 2.

Our main objective was to identify the frequency range within the wave images that has the most effect on the classification results. Our assumption is that if an image segment does not contain a complete cycle of waves at a particular frequency, then the effect of this frequency is unlikely to be significant in the classification results. Controlling the length of data slices can control the range of frequencies the image will include. For example, if $n = 25$, then the time span of that wave image is $0.1s$ and the corresponding frequency is $f > 10\,\text{Hz}$. In short, $n$ determines the lowest frequency that an image would include. To study the impact of partial frequencies at different phases and to augment the data size, we partitioned the wave images with overlapping segments. Such treatment can better reflect the relationship of waves with different frequencies.

**Figure 3:**
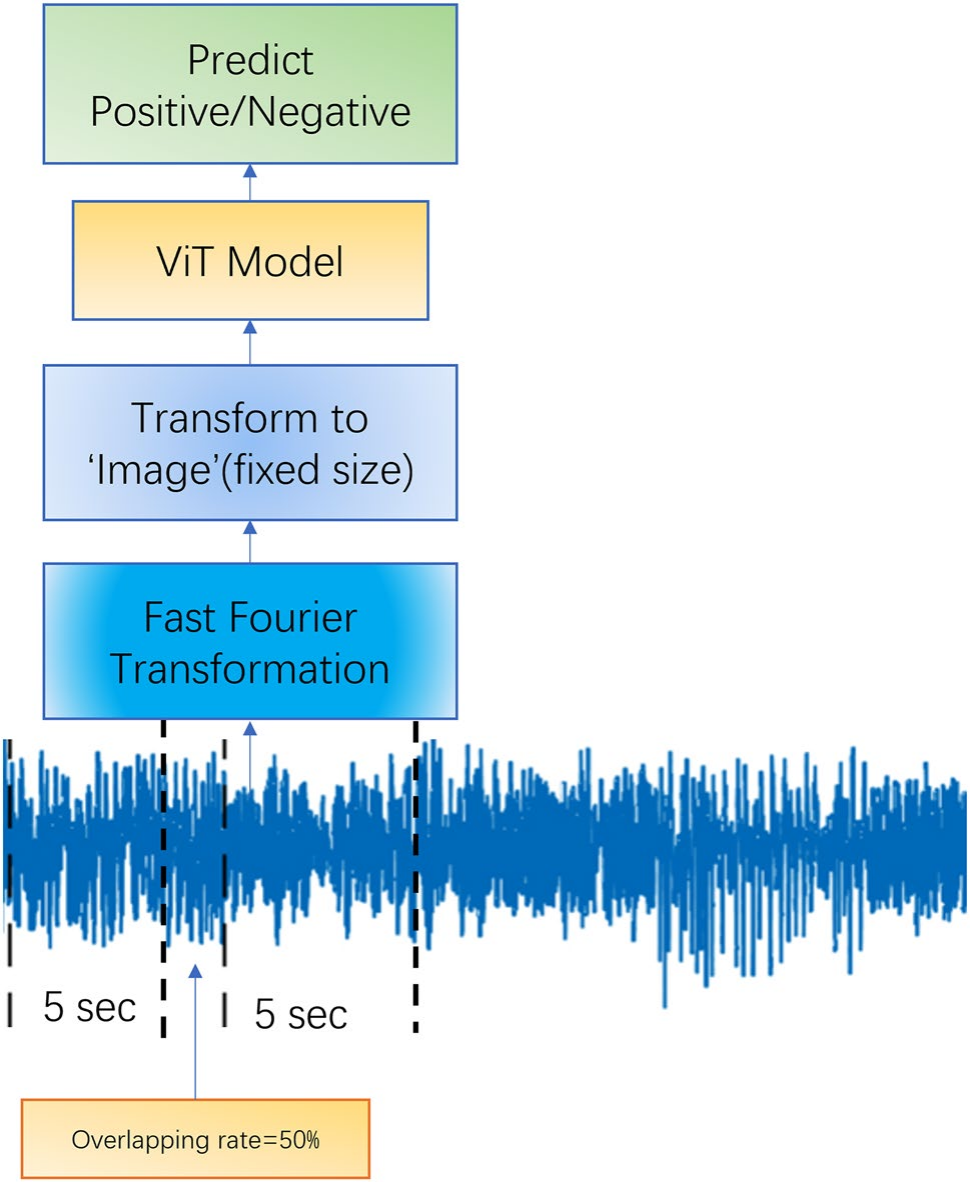
Working pipeline for models trained under frequency domain in comparison experiment.

To understand how the results are related to the sizes of the slices, five different lengths were chosen: twenty-five rows (0.1 s), 125 rows (0.5 s), 250 rows (1 s), 400 rows (1.6 s), and 1250 rows (5 s). Due to the small size of population, data is augmented using an overlapping window scheme, where the starting row of the next data slice is found somewhere in the current data slice, rather than after the last row of the current data slice. An example of 30% overlapping data slice of one second (250 rows) is shown in Fig. 3. Relevant signals in EEG study include the alpha (8–12 Hz), beta (15–30 Hz), delta (0.5–3 Hz), gamma (> 30 Hz), and theta (4–7 Hz) waves. If we use $T$ to represent the time span of each data slice, then the frequency an image can detect is $f = \frac{1}{T}$. In the study, the highest minimum frequency it can detect is $10\,\,\text{Hz}$ when $T = 0.1s$, and the lowest minimum frequency it can detect is $0.2\,\,\text{Hz}$, when $T = 5s$. The data slices are randomly split into testing and training sets while avoiding putting all the data from any one subject into one (training + testing) set. Positive cases and negatives in both the training sets and the testing sets are relatively balanced, with a ratio close to one.

The default hyper-parameters of the model were used in this study: batch size = 64, learning rate = 0.001, depth = 12, and heads = 8. Overlapping ratio of 75%, 90%, and 95% were studied. Since no major differences were discovered among these different overlapping ratios, 90% windows overlapping ratio was used to report the results. EGG data converges very quickly when using ViT model. In most situations, training accuracies achieved more than 99% in as little as three epochs. To avoid overfitting, model trained after 5 epochs were used to evaluate the testing datasets.

The reason for using ViT, a transformer-based CV model instead of transformer-based language model is that the data are a mix of waves of different frequencies. Given that waves are recurring periodically, an image segment containing at least one full cycle of a particular frequency may not provide sufficient information to analyze its data. This study investigated the image segments that contain a collection of waves with different frequencies. Since EEG data are best presented in wave image format, the Transformer based NLP model is not the best suited model to analyze EEG wave images. As such, we decided to employ ViT rather than Transformer to analyze the data.

Note also that it is a customary practice to transfer time series data into spectral images via transformation techniques like fast Fourier transformation (FFT). We argue such transformation is not well suited for the ViT model. In this work, we also studied the effect of adding FFT to the process. The workflow is shown in Fig. 3.

To better understand the value of ViT model in EEG data analyses, a public dataset^28^ were also used to perform a binary classification task. The data slice of 1250 rows was adopted and the overlapping ratio was set to 90%. The model achieved testing accuracy of 86.33%, which is better than state-of-the-art algorithms SleepEEGNet at the accuracy of 80.03%. The pilot results show that ViT is a better fit to analyze EEG data than existing algorithms.

## Results

Fifteen different treatments (5 data slice sizes $\times$ 3 overlapping rate) were used to evaluate the performance of ViT model. Since overlapping rates did not affect the accuracy results, only findings of $over\;lapping\; rate = 90\%$ were reported, see Table 1.

**Table 1:** Results of IDA cleaned data (overlapping rate = 90%).

Time (# of rows in a data slice)	Training accuracy (%)	Testing accuracy (%)
0.1 s (25 rows)	99.99	51.86
0.5 s (125 rows)	99.99	72.82
1 s (250 ross)	99.99	94.99
1.6 s (400 rows)	99.99	95.14
5 s (1250 rows)	99.99	**97.58**

Significant values are in bold.

**Figure 4:**
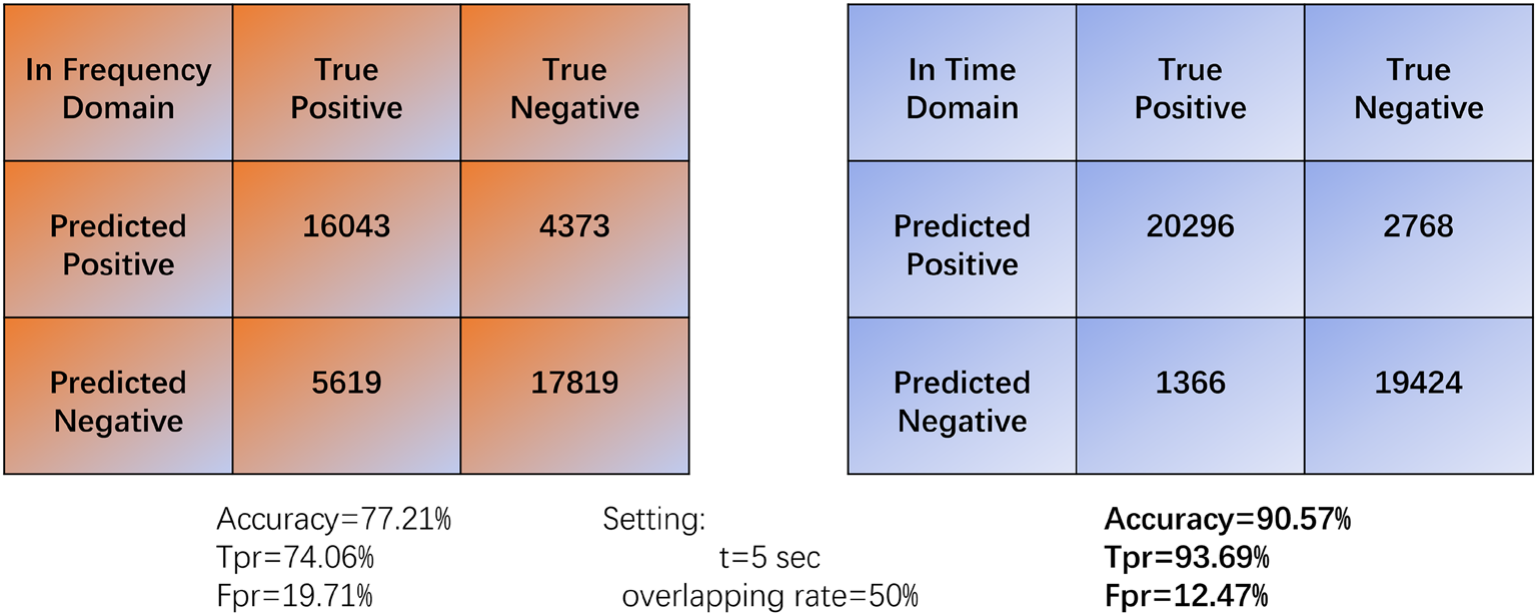
The confusion matrix for the comparison experiment, with the left portion showing the matrix for the model trained in the frequency domain and the right portion depicting the matrix for the model trained in the time domain.

The model reached best testing accuracy of 97.58% when data slice has a size of 1250 rows (5 s). Figure 4 illustrates the impact of data slices on prediction accuracy.

When data slice is as little as twenty-five rows, it contains full cycles of waves that are higher than $10\;{\text{Hz}}$. The prediction accuracy was merely 51.86%, suggesting the ViT did not learn anything useful from the data. The bad results could suggest that waves with higher frequencies are less important when predicting delirium. It could also because the sizes of the data slides were too small to be resized to a $224 \times 224$ images. When a data slice contains at least a full cycle of $2 \;{\text{Hz}}$ waves (0.5 s, or 125 rows), the accuracy increased to 72.82%. When a data slice contains at least a full cycle of $1 \;{\text{Hz}}$ waves (1 s, or 250 rows), the accuracy is further increased to around 95%. Better results are associated with waves with lower frequency. Further investigation is needed to fully understand if long wave signals, such as delta waves, are truly the predictors for delirium. In most of the experiments, the models converged in three epochs. Accuracies did not improve from epoch six to epoch ten.

To study the results of analyzing EEG data in frequency domain, we compared its confusion matrix against the data in time domain. The experiment parameters were $t = 5s$, $overlapping\; rate = 50\%$, and epochs were set to 4. Figure 4 presents the results of this contrastive study. It demonstrates that the model's performance under the time domain outperforms its performance under the frequency domain. These findings align with our discussion and further support the effectiveness of our feature extraction approach in time the domain and selection of the ViT model to analyze EEG data in wave image format.

Since ViT worked well on data cleaned using IDA, it begs for the question: would ViT provide good prediction results on datasets that are not cleaned by IDA? To answer this question, uncleaned data were also fed to the ViT model to evaluate the results. Data slices of 1250 were chosen with an overlapping rate of 90%. To our surprises, both training and testing accuracies reached more than 99.99%. This may suggest that deep learning techniques like ViT probably do not need any additional feature engineering techniques to achieve impressive results. Because the dataset is small, it is too soon to make such a claim. Additional studies are needed to validate this hypothesis.

The datasets collected by the researchers were limited to only twelve subjects and were used only on delirium predictions. What about other EEG datasets? We did another experiment to understand if ViT is still applicable. We also applied ViT in a public EEG data set^28^ to do a binary classification task. Under windows size of 1250 points, the model reached 86.33% test accuracy.

## Discussion

This is the first prospective pilot study using a 10-electrode rapid response EEG device providing data from all lobes in the cerebrum, supervised deep learning and ViT to evaluate EEG for the presence of delirium. This pilot established that such monitoring is feasible in critically ill older adults across medical, surgical, and cardiac ICUs. The principal finding is that using supervised deep learning and a ViT platform, patients were accurately classified as delirium positive or delirium negative based on identifiable characteristics in EEG thus predicting the presence of delirium. These results were replicated using three methods of machine learning, supervised learning methods included stepwise linear discriminant analysis and support vector machines and supervised deep learning was conducted using ViT. Compared to prior studies using various machine learning and preprocessing methods, the ViT model using the hyperparameters mentioned above has provided greater accuracy. For example, van Sleuwen and Sun^29^ used a 3-channel limited lead device to measure physiologically based methods using the Confusion Assessment Method-Severity Score. To obtain three channels or waveforms, they used 6-s EEG strips obtained from a 4-electrode frontal montage. Using this montage on 252 delirious and 121 non-delirious patients, they obtained accuracies of 0.63–0.73 on the ROC curve meaning the model accurately predicted 63–70% of true positives for every possible decision threshold of the model. Similarly, Yamanashi and Kajitani^30^ was able to obtain AUCs of 63–76% using a bispectral EEG with two channels. As a result, they recommended further studies may benefit from deep learning models such as the one used in this pilot study.

The merit of this handheld rapid response EEG is its objectiveness compared to currently available bedside screening methods. Additionally, this method does not require the use of a large EEG machine and specialized technicians for electrode placement that frequently limit large volume mass screening such as that needed to screen for delirium in critical care units. Rapid response EEG is easy to use by busy hospital staff with minimal training. Use of pre-programmed algorithms, such as the one described here, limits the need for skilled interpretation required when using traditional EEG. Therefore, this method of detection is not limited by subjectivity and rationalizing of results associated with bedside screening.

While the ViT model used above has satisfactory performance there are still opportunities to improve prediction. For example, using a window of 10–60 s instead of the 1–5 s windows typically used in this type of analysis may have provided more data points. During the pre-processing phase, our study used sequence extraction meaning samples are obtained in a chronological sequential order and finite length. It is possible that constant correction or interpolation (removing data obtained from lead electrode that is bad) may provide a cleaner data set for analysis.

### Limitations

Limitations of this study include the small sample of thirteen participants with seven experiencing delirium during the monitoring period as determined by the CAM-ICU. EEG changes occur prior to symptom onset and therefore, some of the participants may have had subsyndromal delirium detected using EEG that were not detected using bedside screening (CAM-ICU). Delirium assessments were conducted by the researcher rather than using clinician assessments providing stronger reliability of delirium status. Heterogeneity of the sample with varying etiologies and medication exposures could have resulted in some EEG changes being reflected more than others in a subset of patients, thus minimizing generalizability. While this study has limitations, consistent results have been obtained across methods of analysis (frequency ratios, supervised learning, and deep learning) providing strength to the findings.

## Conclusions

In this analysis, we trained a ViT model to analyze EEG data under the constraint of a small sample and therefore limited amount of data. Despite the use of a sequence extraction method for preprocessing, 97% accuracy is significantly better than the ~ 40% accuracy of clinician derived CAM-ICU assessments. Therefore, this method has strong potential for improving accuracy of delirium detection, providing greater opportunity to implement and evaluate individualized interventions. Once early detection of brain dysfunction associated with poor outcomes such as the need for institutionalization and higher mortality are readily available, it will be possible to identify reversible causes, followed by early intervention and close monitoring to avoid preventable complications. Having a physiologic method for delirium detection may provide an opportunity to provide interventions when delirium is more amenable to treatment Earlier intervention may shorten the hospital length of stay, increase a patient’s chance to go home after hospital discharge, decrease mortality rates, and reduce the financial burden associated with delirium.

## Data Availability

The datasets generated and/or analyzed during the current study are not publicly available but are available from the corresponding author on reasonable request.
